# Effect of Laparoscopic Sleeve Gastrectomy on Calcium Metabolism Parameters, Vitamin D, and Parathyroid Hormone in Morbidly Obese Patients: Short-Term Results

**DOI:** 10.7759/cureus.71795

**Published:** 2024-10-18

**Authors:** Nail Omarov, Elnur Huseynov

**Affiliations:** 1 General Surgery, Yeni Yuzyıl University, Istanbul, TUR; 2 General and Obesity Surgery, Gaziosmanpasa Hospital, Istanbul, TUR; 3 General and Obesity Surgery, Avrupa Safak Hospital, Istanbul, TUR

**Keywords:** bariatric surgery, calcium metabolism, parathyroid hormone, sleeve gastrectomy, vitamin d

## Abstract

Objective: Sleeve gastrectomy (SG) is a widely utilized bariatric surgical procedure. It may affect various aspects of nutritional and metabolic health, including calcium (Ca) metabolism, vitamin D levels, and parathyroid hormone (PTH) secretion. We aimed to investigate the effect of laparoscopic SG (LSG) on Ca metabolism parameters, vitamin D, and PTH in morbidly obese patients.

Materials and methods: This single-center, retrospective cohort study was conducted at the Department of General Surgery, Avrupa Safak Hospital, Istanbul, between March 2021 and November 2023. A total of 78 patients were included in the study. The procedure involved the resection of approximately 80% of the stomach, creating a tubular gastric sleeve. All patients underwent postoperative follow-up at six and 12 months.

Results: Of the 78 patients, 49 (62.8%) were females and 29 (37.1%) were males. The mean age of the patients was 31.2±9.8 years. The mean weight was 126±12.2 kg and the mean BMI was 47±6.9 kg/m2. There was a significant decrease in the BMI values following surgery (*p*<0.01). Vitamin D level was found to be 18.7±4.2 ng/mL at baseline, which increased to 37.6±2.5 ng/mL at 12 months after surgery (*p*<0.01). Before surgery, the PTH level was 69.1±8.7 pg/mL, which decreased to 39.3±5.5 pg/mL at 12 months postoperatively (*p*<0.01). The mean serum Ca level at baseline was 9.2±1.4 mg/dL, which increased to 10.1±0.4 mg/dL at 12 months after operation (*p*<0.01).

Conclusion: Our study results suggest that low vitamin D levels are commonly observed in patients undergoing bariatric surgery. Administering vitamin D as a standard postoperative treatment regimen may be helpful in reducing this deficiency. We, therefore, recommend continuous monitoring of vitamin D, PTH, and serum Ca levels after surgery to manage these issues effectively.

## Introduction

Currently, bariatric surgery stands as a cornerstone in the treatment of obesity [1]. Sleeve gastrectomy (SG) is a widely utilized bariatric surgical procedure to address morbid obesity by reducing the stomach's size, thereby limiting food intake and promoting weight loss. While SG is effective in improving metabolic outcomes and reducing obesity-related comorbidities, it may affect various aspects of nutritional and metabolic health, including calcium (Ca) metabolism, vitamin D levels, and parathyroid hormone (PTH) secretion [2]. Malabsorptive procedures, bypassing the stomach and duodenum, have been shown to be associated with a higher degree of deficiencies than restrictive ones [3].

Calcium metabolism is a crucial physiological process, involving the absorption, utilization, and regulation of Ca, which is essential for bone health, muscle function, and various metabolic functions. Following SG, patients often experience alterations in Ca metabolism, which can be attributed to alterations in gastric anatomy and the subsequent impact on nutrient absorption [4]. Postoperative Ca deficiency is a common concern, potentially leading to osteopenia or osteoporosis if not adequately managed [5]. Vitamin D, which plays a major role in Ca absorption and bone health, may also be affected by SG. Of note, vitamin D deficiency is prevalent among individuals undergoing SG, partly due to decreased intake and absorption, as well as changes in the metabolism of the vitamin [6]. This deficiency can exacerbate Ca-related issues and contribute to alterations in PTH levels. Parathyroid hormone is a key regulator of Ca homeostasis. Increased PTH levels following SG may indicate an adaptive response to reduced Ca levels or impaired Ca absorption. In addition, elevated PTH may be a marker of secondary hyperparathyroidism, which may result from impaired Ca and vitamin D metabolism after surgery [7].

In the present study, we aimed to investigate the effect of laparoscopic SG (LSG) on Ca metabolism parameters, vitamin D, and PTH in morbidly obese patients.

## Materials and methods

This single-center retrospective cohort study was conducted at the Department of General Surgery, Avrupa Safak Hospital, Istanbul, between March 2021 and November 2023. A written informed consent was obtained from each patient for all diagnostic and therapeutic procedures. The study protocol was approved by the Istanbul Yeni Yuzyil University Ethics Committee (Approval No. 2024/09-1242).

Inclusion criteria were established in accordance with the American Society for Metabolic and Bariatric Surgery (ASMBS) guidelines as follows: age between 18 and 65 years; body mass index (BMI) ≥40 kg/m2 or BMI ≥35 kg/m2 with obesity-related comorbidities; having no history of gastrointestinal diseases affecting absorption; having no history of chronic diseases affecting vitamin or iron metabolism (e.g., liver disease, chronic renal failure) [8]. Exclusion criteria were as follows: pregnant or lactating women; those receiving medications known to affect vitamin or iron levels (e.g., anticonvulsants, long-term proton pump inhibitors); having prior gastrointestinal surgeries affecting nutrient absorption. A total of 78 patients who met the inclusion criteria were enrolled.

Preoperative assessment

Prior to surgery, all patients underwent a comprehensive preoperative evaluation, including a detailed medical history and physical examination, abdominal ultrasound, upper gastrointestinal endoscopy, functional respiratory tests, nutritional assessment, and dietary history taking. Baseline laboratory tests included serum levels of vitamin D, Ca, and PTH. 

Surgical procedure

All surgeries were performed by a team of experienced bariatric surgeons using standard laparoscopic techniques. The procedure involved the resection of approximately 80% of the stomach, creating a tubular gastric sleeve.

Postoperative follow-up

Following surgery follow-up was carried out by a checking of serum level of 25(OH)D, vitamin D, and PTH. Normal serum level of 25(OH)D was considered >30 ng/mL, vitamin D insufficiency 21 to 30 ng/m, deficiency <20 ng/mL, and severe deficiency <10 ng/mL. Normal PTH level was considered 10 to 65 pg/mL and normal serum Ca level was considered 8.6 to 10.3 mg/dL [9]. All patients were given 400 IU/day of 25(OH)D. If the levels were <30 ng/mL, further doses of calcifediol (200,000 IU) were added every two weeks. 

Statistical analysis

Statistical analysis was performed using the SPSS Statistics for Windows, version 24, (IBM Corp., Armonk, NY, USA). Continuous data were expressed in mean ± standard deviation (SD) or median (min-max), while categorical data were expressed in number and frequency. Independent t‐test and chi‐square test were used to analyze the data. A p-value of <0.05 was considered statistically significant.

## Results

Of the 78 patients, 49 (62.8%) were females and 29 (37.1%) were males. The mean age of the patients was 31.2±9.8 years. The mean weight was 126±12.2 kg and the mean BMI was 47±6.9 kg/m2. The mean hospital stay was 2.1±1.3 days (Table 1). 

**Table 1:** Demographic and baseline characteristics of patients (n=78). SD: Standard deviation.

Variables	n (%)
Sex	
Male	29 (37.1%)
Female	49 (62.8%)
Age (years)	
Mean ± SD	31.2±9.8
Median (min. – max.)	30 (18-52)
Preoperative height (cm)	
Mean ± SD	162.1±6.1
Median (min. – max.)	163 (150-181)
Preoperative weight (kg)	
Mean ± SD	126±12.2 kg
Median (min. – max.)	121 (103-178)
Preoperative vitamin D level	
Deficiency < 20 (ng/ml)	32 (41.02)
Insufficiency 20-30 (ng/ml)	46 (58.9)
Preoperative parathyroid hormone level	
Hyperparathyroidism	12 (15.3)
Normal	66 (84.6)
Preoperative calcium level	
Normal	78 (100)
Hospital stay (day)	
Mean ± SD	2.1±1.3
Early post-operation complication	6 (7.69)
Late post-operation complication	5 (6.4)

There was a significant decrease in the BMI values after surgery (p<0.01). Vitamin D level was found to be 18.7±4.2 ng/mL at baseline, which increased to 37.6±3.5 ng/mL at 12 months after surgery (p<0.01). Preoperative PTH level was 69.1±8.7 pg/mL, which decreased to 39.3±5.5 pg/mL at 12 months postoperatively (p<0.01). The mean serum Ca level at baseline was 9.2±1.4 mg/dL, which increased to 10.1±0.4 mg/dL at 12 months after surgery (p<0.01) (Table 2). 

**Table 2:** Comparison of values preoperative and following SG. SG: Sleeve gastrectomy, BMI: Body mass index, PTH: Parathyroid hormone, a: statistically significant difference in comparison to 6 months, b: statistically significant difference in comparison to 12 months.

Variables	Preoperative Mean ± SD	6 months after SG Mean ± SD	12 months after SG Mean ± SD	P
BMI (kg/m2)	47±6.9	34±5.6	29±4.5	<0.001
Serum 25(OH)D (ng/ml)	18.7^ab^±4.2	21.2^b^±2.9	37.6±3.5	<0.001
Calcium (mg/dl)	9.2^ab^±1.4	9.8^b^±0.9	10.1±0.4	<0.001
PTH (ng/ml)	69.1^ab^±8.7	49.6^b^±7.1	39.3±5.5	<0.001

## Discussion

In recent years, LSG has become a prevalent surgical intervention for morbid obesity, showing significant weight loss and improvement in metabolic parameters. However, concerns have still arisen regarding its impact on Ca metabolism and related parameters, such as vitamin D and PTH levels. There is a growing number of evidence showing that major nutritional deficiencies before surgery in this patient population are low vitamin D, increased PTH levels, low iron, and the presence of anemia [10,11]. It's a given that 90% of morbidly obese patients have low vitamin D levels. [12]. Haghigat N et al. reported a high prevalence of vitamin D deficiency in patients undergoing LSG [13]. This deficiency can be attributed to both reduced dietary intake and impaired absorption of vitamin D due to alterations in gastrointestinal physiology. Vitamin D is essential for Ca homeostasis, and its deficiency may exacerbate conditions related to Ca metabolism. Therefore, postoperative patients are often recommended to increase their vitamin D intake to minimize these risks.

In the current study, the majority of the patients had low 25(OH)D levels preoperatively: 41% presented with vitamin D deficiency, while 59% presented with insufficiency. This may be explained by several factors, including dietary patterns and reduced exposure to sunlight secondary to limited outdoor activities, which is widely considered to be the result of increased uptake in adipose tissue as reported in recent studies [14,15]. Another factor that can lead to impaired nutritional status in patients with obesity is chronic inflammation caused by various proinflammatory cytokines released from adipose tissue [16,17]. In our study, although additional calcifediol supplement was administered, when needed, vitamin D slightly improved, and most patients remained at the level of <30 ng/mL. 

Furthermore, elevated PTH levels are a common finding in the short term after LSG. The PTH levels were negatively correlated with 25(OH)D levels [14], as bone mineral density (BMD) directly correlates with vitamin D and inversely correlates with PTH levels after bariatric surgery [18-20]. The PTH is released in response to low serum Ca levels and is involved in increasing Ca release from bones and enhancing Ca reabsorption in the kidneys. Mendonça F et al. [21] observed increased PTH levels in patients following LSG, indicating the compensatory mechanism of the body to counteract hypocalcemia. The elevated PTH may also indicate secondary hyperparathyroidism, a condition where PTH is elevated due to the body's efforts to maintain normal Ca levels between reduced serum Ca and Vitamin D levels. Persistent elevated PTH may lead to potential bone health issues over time, highlighting the importance of managing Ca and Vitamin D levels effectively [21]. In our study, secondary hyperparathyroidism was observed in 15.3% of patients preoperatively and returned to normal after surgery with vitamin D supplementations. Most of those who had vitamin D insufficiency were found to have secondary hyperparathyroidism. 

Several studies have demonstrated that serum Ca levels may decrease within a couple of months after the procedure. A study by Mayana Alves Baad et al. demonstrated a significant decrease in serum Ca levels within six months after LSG, largely due to decreased Ca intake and absorption inefficiencies resulting from the reduced gastric volume [22]. The smaller gastric pouch and reduced gastric acid secretion can impair the solubilization and absorption of Ca, thereby leading to lower serum levels. Additionally, Ivaska et al. observed that patients exhibited reduced Ca absorption efficiency shortly after LSG [23]. This reduction is compounded by a lower intake of Ca-rich foods and a potential increase in Ca excretion. 

In general, it is recommended to administer vitamin D and Ca to improve BMD in postoperative patients. There are many studies suggesting that patients who undergo LSG should receive substantial amounts of postoperative vitamin D, with a good level of vitamin D being maintained through a weekly dose of 5,000 IU and regular multivitamin maintenance doses both before and after surgery [24,25]. In the current study, we provided higher doses of vitamin D after LSG. To prevent secondary hyperparathyroidism, regular monitoring of PTH and vitamin D levels is also recommended. This allows for the adjustment of vitamin D doses and helps to prevent bone loss related to secondary hyperparathyroidism.

Current guidelines recommend a minimum daily supplementation of 3,000 IU of vitamin D following bariatric surgery. For many patients, particularly those undergoing laparoscopic Roux-en-Y gastric bypass (LRYGB) and LSG, a daily dose of 6,000 IU is considered effective and safe [26].

Nonetheless, there are some limitations to this study. First, the single-center, retrospective design of the study potentially leads to analytic bias. Second, we could only evaluate short-term results. Further large-scale, long-term, prospective studies are warranted to evaluate the long-term effects of LSG on vitamin D and PTH.

## Conclusions

In conclusion, low vitamin D levels are commonly observed in patients undergoing bariatric surgery. Administering vitamin D as a standard postoperative treatment regimen may be helpful in reducing this deficiency and related bone complications. Based on our study findings, we recommend continuous monitoring of vitamin D, PTH, and serum Ca levels after surgery to manage these issues effectively.
